# The co‐location of CD14+APOE+ cells and MMP7+ tumour cells contributed to worse immunotherapy response in non‐small cell lung cancer

**DOI:** 10.1002/ctm2.70009

**Published:** 2024-08-26

**Authors:** Guangyu Fan, Tongji Xie, Le Tang, Lin Li, Xiaohong Han, Yuankai Shi

**Affiliations:** ^1^ Department of Medical Oncology, National Cancer Center/National Clinical Research Center for Cancer/Cancer Hospital, Chinese Academy of Medical Sciences & Peking Union Medical College Beijing Key Laboratory of Clinical Study on Anticancer Molecular Targeted Drugs Beijing China; ^2^ Department of Pathology, National Cancer Center/National Clinical Research Center for Cancer/Cancer Hospital Chinese Academy of Medical Sciences & Peking Union Medical College Beijing China; ^3^ Clinical Pharmacology Research Center, Peking Union Medical College Hospital, State Key Laboratory of Complex Severe and Rare Diseases, NMPA Key Laboratory for Clinical Research and Evaluation of Drug, Beijing Key Laboratory of Clinical PK & PD Investigation for Innovative Drugs Chinese Academy of Medical Sciences & Peking Union Medical College Beijing China

**Keywords:** intra‐tumour immune infiltration, non‐small cell lung cancer, spatial structure, spatial transcriptomics

## Abstract

Intra‐tumour immune infiltration is a crucial determinant affecting immunotherapy response in non‐small cell lung cancer (NSCLC). However, its phenotype and related spatial structure have remained elusive. To overcome these restrictions, we undertook a comprehensive study comprising spatial transcriptomic (ST) data (28 712 spots from six samples). We identified two distinct intra‐tumour infiltration patterns: immune exclusion (characterised by myeloid cells) and immune activation (characterised by plasma cells). The immune exclusion and immune activation signatures showed adverse and favourable roles in NSCLC patients' survival, respectively. Notably, CD14+APOE+ cells were recognised as the main cell type in immune exclusion samples, with increased epithelial‒mesenchymal transition and decreased immune activities. The co‐location of CD14+APOE+ cells and MMP7+ tumour cells was observed in both ST and bulk transcriptomics data, validated by multiplex immunofluorescence performed on 20 NSCLC samples. The co‐location area exhibited the upregulation of proliferation‐related pathways and hypoxia activities. This co‐localisation inhibited T‐cell infiltration and the formation of tertiary lymphoid structures. Both CD14+APOE+ cells and MMP7+ tumour cells were associated with worse survival. In an immunotherapy cohort from the ORIENT‐3 clinical trial, NSCLC patients who responded unfavourably exhibited higher infiltration of CD14+APOE+ cells and MMP7+ tumour cells. Within the co‐location area, the MK, SEMA3 and Macrophage migration inhibitory factor (MIF) signalling pathway was most active in cell‒cell communication. This study identified immune exclusion and activation patterns in NSCLC and the co‐location of CD14+APOE+ cells and MMP7+ tumour cells as contributors to immune resistance.

## Graphical abstract

1. Our analysis identified two distinct infiltration patterns in NSCLC: immune exclusion and immune activation.
2. CD14+APOE+ cells were identified as the predominant cell type in immune exclusion samples.
3. MMP7+ tumour cells were found to co‐localise with CD14+APOE+ cells.
4. NSCLC patients with poor responses to immunotherapy showed higher infiltration of APOE+ TAMs and MMP7+ tumour cells.

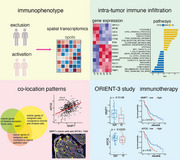

## 1 INTRODUCTION

Cancer immunotherapy has proven to be a highly effective method for combating cancer by harnessing the destructive capabilities of the human immune system. This approach has led to long‐lasting therapeutic improvements, particularly in non‐small cell lung cancer (NSCLC).^1^, ^2^ Despite the significant success of immunotherapy in cancer treatment, tumours can develop various mechanisms of immune resistance.^3^ The tumour microenvironment (TME) encompasses a variety of adaptive and innate immune cells, which may reside within the tumour or be recruited to the site. The interactions between tumour cells and all types of immune cells shape the immunological condition of the tumour, influencing its response to immunotherapy.^4^, ^5^ As tumours progress, the TME gradually becomes more immunosuppressive, characterised by multiple components of the immunosuppressive immune system.^6^, ^7^ These components contribute to the tumour's ability to evade the immune system and resist immunotherapy.^8^

Within the TME, intra‐tumour immune infiltration emerges as a pivotal component that interacts with tumour cells, exerting either pro‐tumour or anti‐tumour effects.^9^, ^10^ Previous research on the TME in NSCLC has mostly concentrated on individual markers or cell types, such as Programmed cell death protein 1 (PD‐1)/Programmed cell death 1 ligand 1 (PD‐L1)/Cytotoxic T‐lymphocyte‐associated protein 4 (CTLA‐4) expression or CD8 T‐cell infiltration, despite the TME's crucial importance; nonetheless, these studies have shown considerable diversity and lack of consensus.^9^, ^11^, ^12^ Furthermore, the characterisation of the TME has historically been subjective, often relying on immunohistochemistry (IHC) staining, bulk transcriptomics data and single‐cell transcriptomics data, without a comprehensive exploration of cell composition and interactions.^10^, ^13^, ^14^ Moreover, the complex relationship between tumour cells and the immune system inside the TME has not been fully understood, despite the considerable influence that tumour cells exert on shaping the immunophenotype of the TME. A more nuanced understanding of these dynamics is essential for advancing our comprehension of immunotherapy and enhancing therapeutic strategies targeting the TME.

Bulk RNA sequencing eliminates the differences in tissue context and gives a combined expression profile of different cell types, such as epithelial, endothelial, stromal and immune cells, in the specific tissue. The implementation of single‐cell RNA sequencing has made it possible to thoroughly analyse the variability in gene expression at the level of individual cells. This technique allows for the identification and understanding of the communication networks between cells in the TME.^15^, ^16^ Recent advancements in spatial transcriptomic (ST) technologies have offered powerful tools for profiling the precise spatial distribution of genes and deciphering the composition of the TME, which affects tumour development and therapeutic response.^17^, ^18^, ^19^ ST, by preserving tissue architecture, allows for the examination of the molecular and cellular composition of intra‐tumour immune infiltration, as well as interactions with neighbouring components of the TME.^20^, ^21^

In this study, we employed ST to analyse and describe the characteristics of immune cell infiltration inside the TME of NSCLC. We delineated two distinct infiltration patterns: immune activation and immune exclusion. We identified plasma cells as the predominant cell type in immune activation samples, whereas CD14+APOE+ cells were prevalent in immune exclusion samples. The spatial co‐location of CD14+APOE+ cells and MMP7+ tumour cells was observed in ST data and further validated through multiplex immunofluorescence analysis conducted on 20 NSCLC samples. Both CD14+APOE+ cells and MMP7+ tumour cells were associated with poorer survival outcomes and immunotherapy therapy response, underscoring their potential as therapeutic targets. To summarise, our study provides insights into the composition of immune cells within tumours in NSCLC and emphasises the presence of CD14+APOE+ cells and MMP7+ tumour cells in close proximity as significant factors in immune exclusion.

## 2 RESULTS

### 2.1 Identifying cancer cells in spatial transcriptomics data

To investigate the spatial organisation of NSCLC, ST sequencing was conducted on the Formalin‐fixed paraffin‐embedded (FFPE) tissue blocks from six NSCLC patients. This process yielded a median of 4796 spots per sample, with each spot containing an average of 5697 genes. The proportion of mitochondrial genes was less than 5% in all samples, and comprehensive quality information is provided in Table S1.

Determining the presence of cancer cells in ST data is challenging, particularly when distinguishing cancer cells from normal epithelial cells. In order to tackle this situation, inferCNV analysis was conducted to distinguish malignant cells from other cell types by examining their copy number variation (CNV) patterns. This procedure consisted of two clustering stages.

**FIGURE 1:**
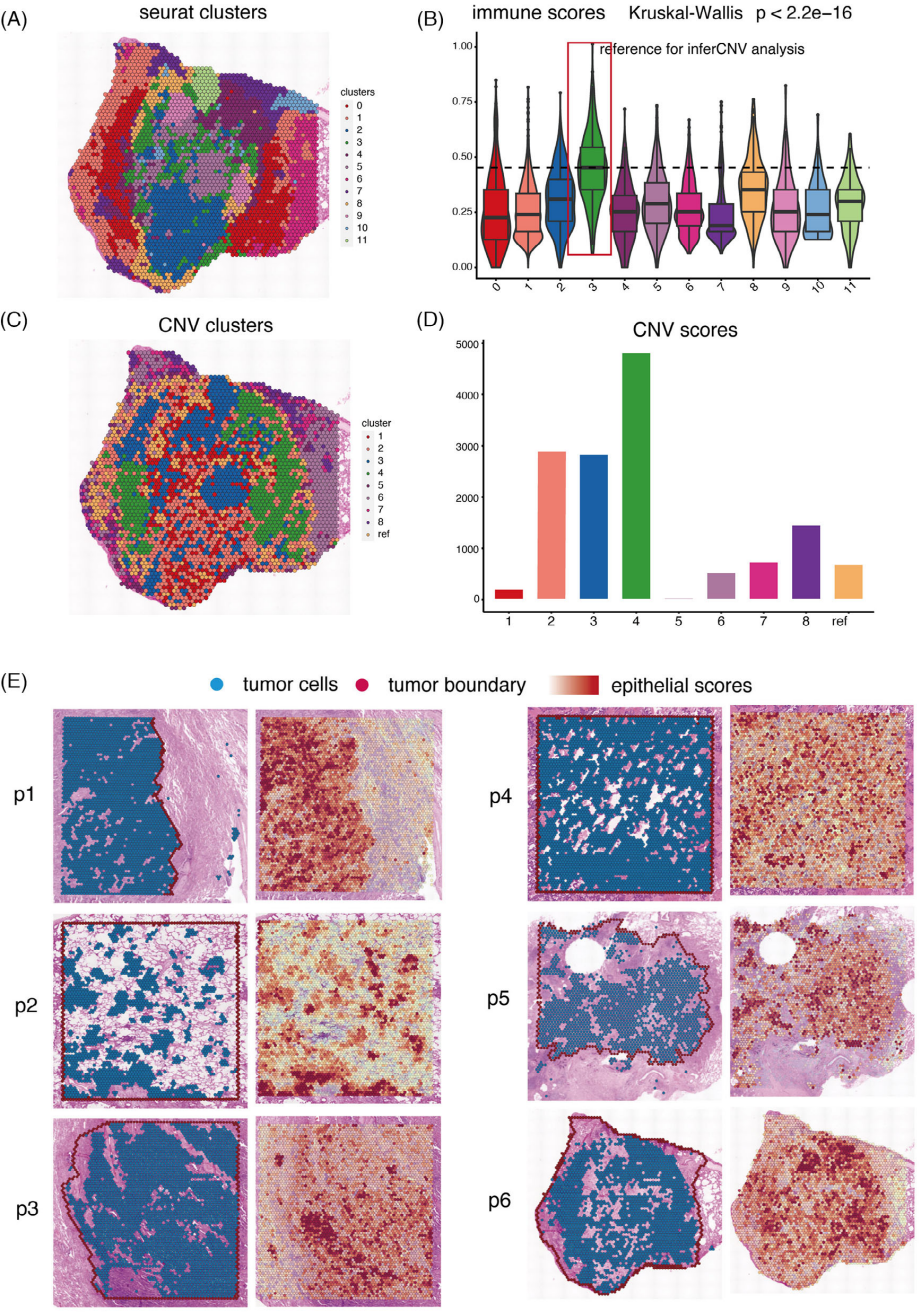
The identification of malignant cells in spatial transcriptomics (ST) data. (A) Clustering of 4796 spots in patient 6 (p6) into 11 distinct clusters. (B) Distribution of immune score including pan‐immune markers (PTPRC), pan‐T‐cell markers (CD2, CD3D, CD3E and CD3G), B‐cell markers (CD79A, MS4A1 and CD79B) and myeloid cell markers (CD68 and CD14) in the 11 clusters. (C) Hierarchical clustering assigning all spots in p6, except the reference cluster, into eight clusters. (D) Bar charts showing the distribution of copy number variation (CNV) score in the nine clusters. (E) The plot depicted the identified tumour cells and the distribution of tumour‐specific epithelial score in six tumour samples.

In the preliminary phase, we determined the reference cells to be used in the inferCNV pipeline. Initially, all regions were segregated into smaller clusters based on gene expression patterns. The ‘immune score’ was calculated for each area using a panel of immune‐related markers, including pan‐immune markers (PTPRC), T‐cell markers (CD2, CD3D, CD3E and CD3G), B‐cell markers (CD79A, MS4A1 and CD79B) and myeloid cell markers (CD68 and CD14). These markers were employed to evaluate the mean degree of immune infiltration within every spot was compared among different clusters using the Kruskal‒Wallis rank sum test. The cluster with the highest immune score was selected as the reference for the inferCNV analysis. Patient 6 (p6) had a total of 4473 spots that were divided into 11 distinct clusters. Out of all the clusters, cluster 3 exhibited the highest immune score and was selected as the reference cluster for inferCNV analysis (Figure 1A,B).

In the second phase of clustering, hierarchical clustering was employed to divide the spots into eight clusters, excluding the reference cluster. Clusters 2, 3 and 4, characterised by significantly elevated CNV values, were identified as malignant clusters, while the remaining clusters exhibited considerably lower CNV scores (Figure 1C,D). The accuracy of these annotations was verified by consulting two distinct pathologists who analysed the histology material acquired from haematoxylin‒eosin staining (HE). Clusters 2, 3 and 4 indicated dispersed tumour regions, while the other clusters primarily consisted of normal epithelial cells, stromal cells and a mixture of immune cells.

Subsequently, the same pipeline was applied to additional samples, identifying tumour areas in each sample (Figure 1E). The identified tumour cells were confirmed to be within the tumour areas annotated by HE histology information. Our team pathologist utilised the Cloupe software developed by 10× Genomics to accurately mark the tumour boundaries based on HE staining of FFPE tissue. Within these boundaries, some regions without tumour cells consist of intra‐tumour fibroblasts and immune cells. Conversely, tumour cells found outside these boundaries are indicative of scattered tumour cells invading the normal tissue. Additionally, calculation of tumour‐related epithelial markers (EPCAM, KRT8 and KRT19) for each spot in all samples revealed that tumour areas showed the highest epithelial scores compared to other areas, confirming the accurate identification of tumour areas (Figure 1E).

### 2.2 Immune activation and immune exclusion patterns in NSCLC

**FIGURE 2:**
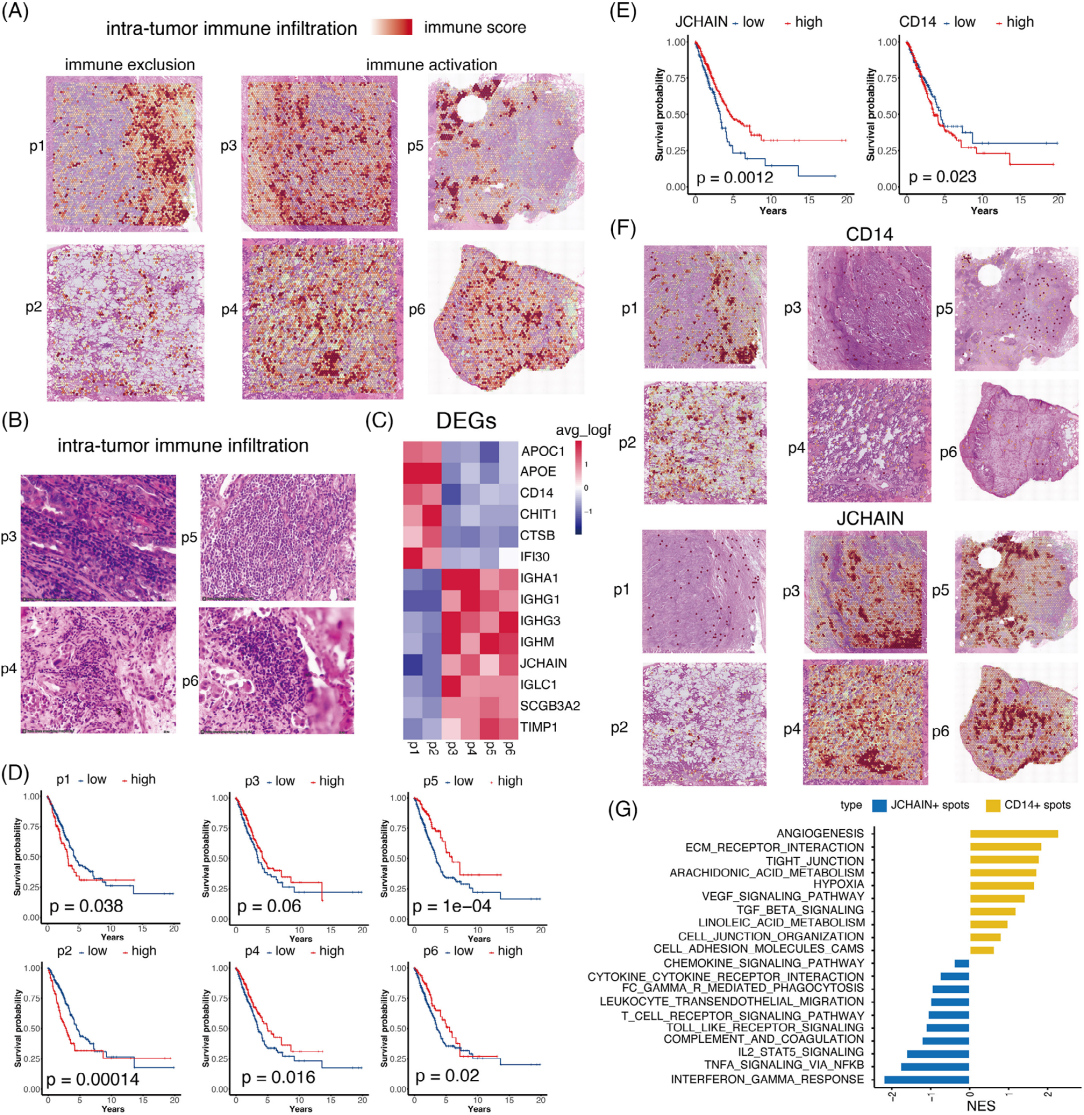
Immune activation and immune exclusion patterns in non‐small cell lung cancer (NSCLC). (A) The plot depicted the distribution of immune score in six NSCLC samples. (B) The corresponding haematoxylin‒eosin staining (HE) images of p3 to p6 displayed the infiltrated immune clusters observed from spatial transcriptomics (ST) data. (C) The differentially expressed genes (DEGs) of the immune clusters in p3 to p6, respectively. (D) The role of infiltrated immune signature (top 50 DEGs) of p3 to p6 in patients’ survival, respectively. (E) CD14 as the macrophage marker and JCHAIN as the plasma cell marker showed the adverse and favourable role in NSCLC patient’ survival, respectively. (F) Distribution of the macrophage marker CD14 and the plasma cell marker JCHAIN. (G) The enriched pathways in CD14+ and JCHAIN+ spots.

To explore the immune infiltration landscape in NSCLC, we evaluated the immune score in each sample, which represents the average level of immune infiltration (Figure 2A). Notably, two samples (p1 and p2) displayed the absence of immune cells within the tumour region, while the remaining four samples showed significant intra‐tumour immune infiltration. The corresponding HE images corroborated the observed immune infiltration patterns in the ST data, confirming the presence of immune cell clusters within the tumour area (Figure 2B).

Previous studies have suggested that intra‐tumour immune components interacting with malignant cells contribute to tumour progression, relapse or metastasis.^22^, ^23^ Subsequently, we investigated the specific cell composition of intra‐tumour immune clusters in samples with distinct intra‐tumour immune infiltration. Using the Cloupe software developed by 10×, we manually extracted the immune clusters in each sample and identified their differentially expressed genes (DEGs) compared to other clusters within the same sample (Figure 2C). The DEGs were used to characterise the immune infiltration clusters in our study. While it is true that these DEGs are specific to each patient, our intention was to identify the composition of immune activation or exclusion that could be generalised across patients. The identification of these clusters was crucial to understand the broader immune landscape within the TME. Plasma cell‐related markers (IGHA1, IGHG1, IGHG3 and JCHAIN) exhibited significantly high expression in samples with immune activation. Recent studies have found that patients treated with immunotherapy for several types of malignancies, such as NSCLC sarcoma, renal cell carcinoma and melanoma, have shown improved clinical outcomes with increased plasma cell signatures.^24^, ^25^, ^26^ Furthermore, the existence of tertiary lymphoid structures and organised lymphoid aggregates is correlated with B cells and plasma cells.^27^, ^28^ In addition, immune exclusion samples exhibited elevated expression of CD14, which is a well‐established marker for myeloid cells. CD14+ cells play a crucial role in various immunosuppressive processes. They are involved in the detection of tumour antigens, the recruitment and functioning of immunosuppressive cells, and the release of immunosuppressive cytokines.^29^, ^30^

To assess the impact of these immune infiltration clusters on survival, we utilised the top 50 DEGs of these clusters in each sample as the signature (Table S2). In a large‐scale transcriptomics cohort obtained from The Cancer Genome Atlas (TCGA), the immune exclusion signature identified in samples p1 and p2 had a negative impact on patient survival. Conversely, patients with an immune activation signature observed in samples p3 to p6 had a longer overall survival (OS), as shown in Figure 2D. JCHAIN, serving as the plasma cell marker, demonstrated a favourable role in NSCLC patients' survival, consistent with the immune activation signature from p3 to p6 (Figure 2E). In contrast, the presence of CD14 was linked to negative outcomes in the survival of patients with NSCLC (Figure 2E). Subsequently, we analysed the spatial distribution of JCHAIN and CD14 expression patterns. We examined the spatial distribution of CD14 and JCHAIN expression within the TME. Our results showed that regions with high CD14 expression were often areas of immune exclusion, while regions with high JCHAIN expression were indicative of immune activation (Figure 2F). We analysed CD14+ and JCHAIN+ spots across six samples and found that pro‐tumour pathways, including angiogenesis, extracellular matrix (ECM) receptor interaction and Vascular endothelial growth factor (VEGF) signalling, were enriched in CD14+ spots. Conversely, pro‐inflammatory pathways, such as interferon gamma response and complement activation, were enriched in JCHAIN+ spots (Figure 2G).

### 2.3 CD14+APOE+ cells was the primary immune cell type in immune exclusion samples

**FIGURE 3:**
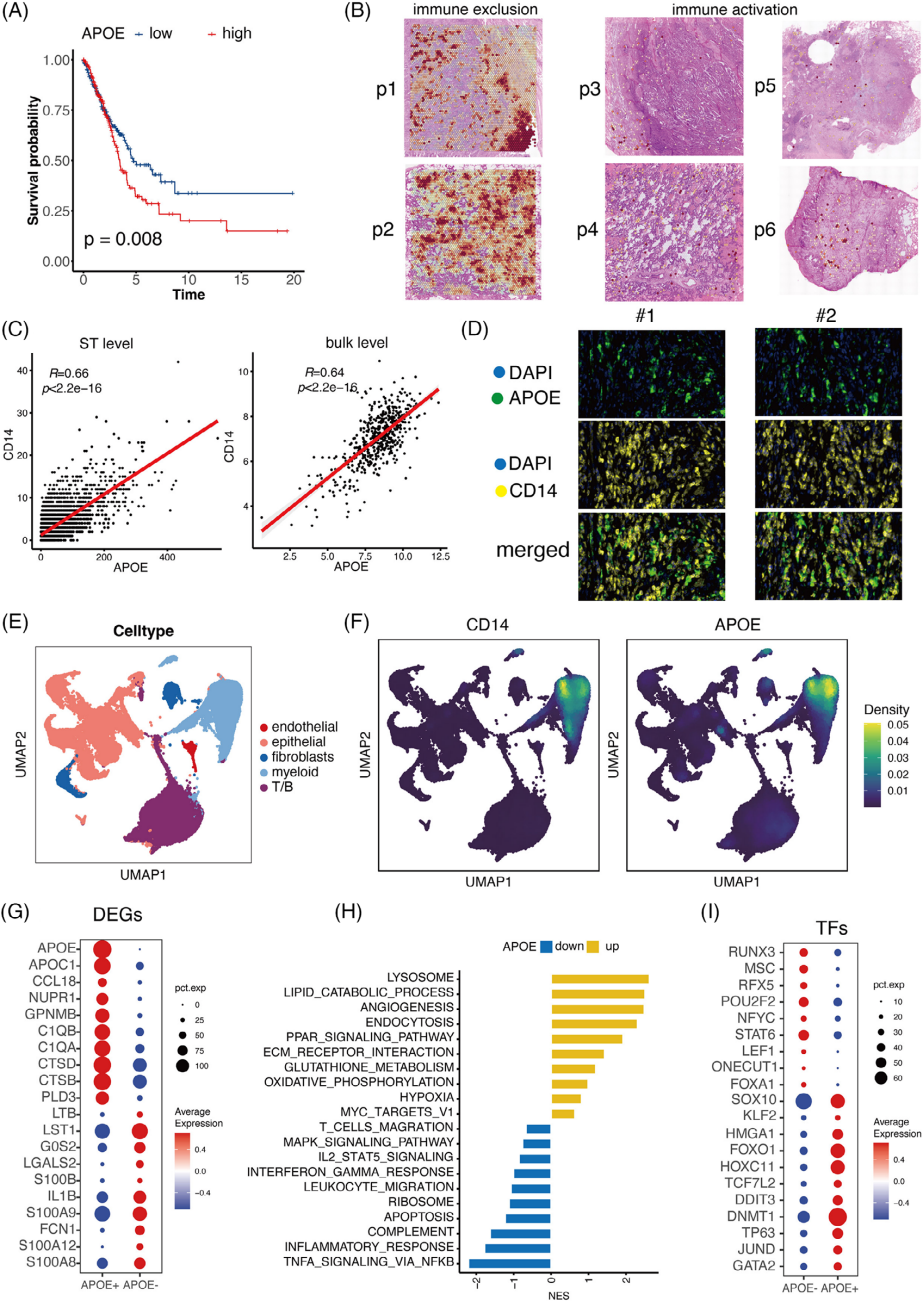
APOE+ tumour‐associated macrophages (CD14+ cells) was the primary immune cell type in immune exclusion samples. (A) Non‐small cell lung cancer (NSCLC) patients with high APOE levels had shorter overall survival (OS) time. (B) Distribution of APOE in samples with distinct immune infiltration patterns. (C) The plot displayed the correlation between APOE and CD14 in the spatial transcriptomics (ST) and bulk transcriptomics data. (D) The multiplex immunofluorescence performed in 20 NSCLC patients demonstrated the co‐location between APOE and CD14. (E) The single‐cell dataset was divided into five main clusters: epithelial cells, myeloid cells, fibroblasts, endothelial cells and T/B cells. (F) Distribution of CD14 and APOE in single‐cell data. (G) The top 10 upregulated genes in APOE+ and APOE‒ CD14+ cells. (H) Bar chart displaying the upregulated and downregulated pathways in CD14+APOE+ cells. (I) Expression patterns of the most varied transcription factors (TFs) in CD14+APOE+ cells.

Within immune exclusion samples, APOE was discovered to have a significant level of expression in intra‐tumour immune infiltration. NSCLC patients who had elevated levels of APOE had a lower OS time, suggesting that APOE has a negative impact on clinical outcomes (Figure 3A). APOE exhibited substantial expression in the tumour region of immune exclusion samples, while it displayed low levels of expression in immune activation samples (Figure 3B). The correlation coefficient between APOE and CD14 was found to be.66 in ST data and.64 in bulk transcriptomics data. This indicates that APOE is a reliable subtype marker for CD14+ cells in NSCLC, as shown in Figure 3C.

In order to confirm the presence of APOE at the protein level, we conducted multiplex immunofluorescence on a group of 20 NSCLC patients. CD14 and APOE were found to co‐localise, suggesting that APOE can serve as a marker for CD14+ cells (Figure 3D). we utilised the software QuPath to perform detailed and quantitative analysis of stained tissue sections. We imported the images into QuPath and performed cell segmentation by detecting cell nuclei, which is crucial for accurate cell counting and segmentation. We then identified cells exhibiting positive staining for specific markers, including PanCK (tumour marker), CD14 and APOE. Detection parameters such as threshold, minimum and maximum cell size, and staining intensity thresholds were set to default values to ensure consistency across all samples. We measured the staining intensity of PanCK, CD14 and APOE within the detected cells and calculated the correlations between these markers. The correlation between PanCK and CD14/APOE was −.085/.098, indicating no significant relevance of CD14/APOE with tumour cells (Figure S1). The correlation between CD14 and APOE was.76, indicating a strong association between these markers (Figure S1). This strong correlation suggests that APOE may serve as a subtype marker for CD14+ cells. The quantification demonstrates the co‐localisation of CD14 and APOE at the protein level, strengthening our conclusion that APOE is associated with CD14+ cells.

To accurately investigate the phenotype of APOE, we collected multiple single‐cell datasets (comprising GSE148071, GSE127465 and GSE131907) and identified five main clusters: epithelial cells, endothelial cells, fibroblasts, myeloid cells and T/B cells^31^, ^32^, ^33^ (Figure 3E). The distribution of APOE exclusively in CD14+ cells validated its role as a typical macrophage marker and represented a subset of CD14+ CD14+ cells (Figure 3F). By comparing APOE+ and APOE‒ CD14+ cells, we compiled a list of DEGs (Figure 3G). Among these DEGs, APOC1, a lipid‐related protein, has been reported to be associated with immunosuppressive macrophages, establishing premetastatic niches and facilitating metastasis in the immunosuppressive TME.^34^ Additionally, C1QB and C1QA were identified as markers of immunosuppressive M2 macrophages and were found to be substantially expressed in CD14+APOE+ cells, related with a lower number of T cells and increased malfunction of T cells.^35^, ^36^ CTSD and CTSB, two aspartic proteases, have been linked with tumourigenesis and worse therapy response in breast cancer and glioblastoma.^37^, ^38^

Pathway analysis revealed an increased lipid catabolic process in CD14+APOE+ cells (Figure 3H), along with enhanced angiogenesis, ECM receptor interaction and hypoxia. The CD14+APOE+ cells exhibited upregulation of glutathione metabolism and the PPAR signalling pathway. Conversely, immune‐related pathways such as TNFA signalling, T‐cell migration, complement and inflammatory response were suppressed in CD14+APOE+ cells. Furthermore, we scrutinised the role of transcription factors (TFs) in promoting the aggressive phenotype of CD14+APOE+ cells (Figure 3I). Significant discoveries revealed increased regulon activities of SOX10, a protein that facilitates the transformation of cutaneous melanoma cells into a condition that is tolerant to targeted inhibitors.^39^ Additionally, the KLF2 maintaining tumour cells survival, exhibited heightened regulon activities in CD14+APOE+ cells.^40^, ^41^ HMGA1 also displayed increased activities and was linked with epithelial‒mesenchymal transition (EMT) and cancer cell stemness through transcriptional and post‐transcriptional interactions.^42^

### 2.4 MMP7 was highly expressed in tumour area with infiltrated CD14+APOE+ cells

Tumour cells have been proven to restructure the immunological elements of the TME to facilitate immune evasion, tumour growth, metastasis and therapeutic resistance. Thus, we conducted a more in‐depth analysis of the characteristics of the tumour cells in samples with different levels of immune infiltration. Our goal was to identify potential interactions between the tumour and the immune system that could be targeted for immunotherapy in NSCLC.

**FIGURE 4:**
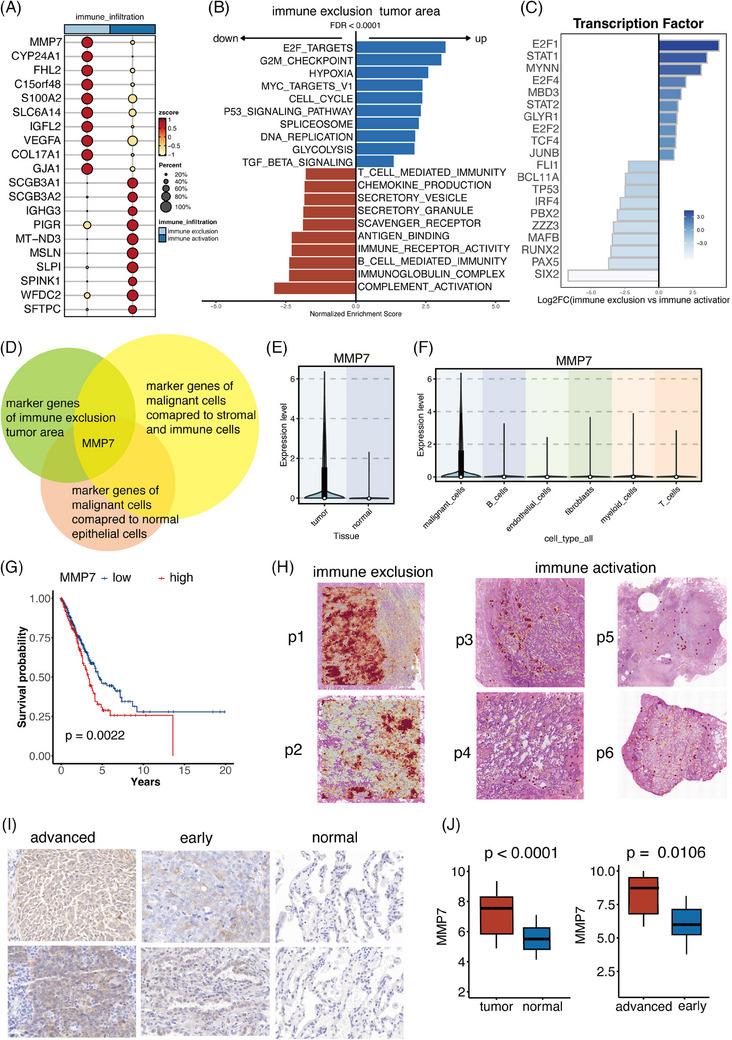
MMP7 was highly expressed in tumour area with infiltrated APOE+ tumour‐associated macrophages (CD14+ cells). (A) The differentially expressed genes (DEGs) of tumour cells in samples with distinct immune infiltration patterns. (B) Pathway analysis unveiled distinct biological activities in immune activation and immune exclusion tumour areas. (C) Expression patterns of the most varied transcription factors (TFs) in two types of tumour areas. (D) MMP7 was identified as the potential tumour marker correlating with the infiltrated CD14+APOE+ cells. (E) MMP7 exhibited elevated expression in tumour cells compared to normal epithelial cells. (F) MMP7 exhibited elevated expression in tumour cells compared to immune cells and stromal cells. (G) Patients with high MMP7 levels had shorter survival time. (H) The spatial expression patterns of MMP7 in two immune infiltration types of samples. (I) Immunohistochemistry images of MMP7 in our cohort consisting of 60 non‐small cell lung cancer (NSCLC) patients. (J) Boxplots displaying the distribution of MMP7 in normal, early and advanced stage samples.

First, we calculated the DEGs of the tumour area in samples with distinct immune infiltration patterns (Figure 4A and Table S3). One of the DEGs found in the immune exclusion samples is MMP7. MMP7 has a role in facilitating tumour growth by stimulating ECM degradation, migration and invasion of tumour cells, and angiogenesis.^43^ VEGFA exhibited upregulation in the tumour area with immune exclusion, stimulating angiogenesis and facilitating tumour growth and metastasis.^44^ SLC6A14 was found in the tumour area with immune exclusion, associated to amino acid supplementation, promoted EMT‐induced metastasis in cancer.^45^ COL17A1, a transmembrane collagen protein, facilitated tumour growth and predicted poor prognosis in pancreatic cancer and regulated dormancy in human colon cancer stem cells.^46^ In the tumour activation samples, SCGB3A1 and SCGB3A2, were highly expressed. These multifunctional cytokine‐like molecules exhibit anti‐inflammatory, growth factor, anti‐fibrotic and anti‐cancer activities, impacting various lung diseases. Polymeric immunoglobulin receptor (PIGR) was highly expressed in immune exclusion samples, playing a crucial role in the immune system by facilitating the transport and secretion of immunoglobulins across epithelial cells. Secretory leukocyte protease inhibitor (SLPI) showed upregulation in the tumour area with immune activation, contributing to the transport of class‐switched immunoglobulin G (IgG) and IgA antibodies.

Pathway analysis revealed distinct biological activities in tumour areas (Figure 4B). The tumour area with immune exclusion was characterised by the activation of pathways related to cell proliferation, including the E2F targets, cell cycle and G2M points. Increased hypoxia activities were observed in the tumour area with low immune infiltration, along with enhanced activity of the well‐known immunosuppressive Transforming growth factor beta (TGFB) pathway and glycolysis. Conversely, the tumour area with high intra‐tumour immune infiltration exhibited enhanced immune responses, including complement activation, B‐cell‐mediated immunity, chemokine production and antigen binding. Additionally, increased secretory activities were observed, including secreting vesicles and granules.

Furthermore, we scrutinised the role of TFs in promoting the immune exclusion phenotype (Figure 4C). The expression patterns of the top 20 TFs with the most varied activities in cellular populations are illustrated. Prominent discoveries revealed heightened regulon activities of the E2F family (E2F1, E2F4 and E2F2), which are crucial in promoting tumour growth and migration.^47^ In the immune exclusion tumour area, STAT1 and STAT2 from the STAT family showed increased regulon activities, which contribute to the promotion of proliferation, survival, angiogenesis and immunological escape.^47^, ^48^ MYNN also exhibited heightened activity, resulting in an enhanced susceptibility to colorectal cancer and bladder cancer.^49^

We further aimed to identify tumour‐specific genes that induce CD14+APOE+ cells infiltration and exert immunosuppressive effects on the tumour TME. Using multiple single‐cell datasets, we identified certain genes that were unique to tumour cells in comparison to immune cells and normal epithelial cells. We determined these genes based on two criteria: an average log2 fold change (avg_log2FC) greater than.25 and a *p*‐value less than.05. This information is illustrated in Figure 4D. By intersecting these three gene lists, we have identified MMP7 as meeting the criteria and exhibiting the highest level of expression in tumour sites characterised by immune exclusion (Figure 4D). Figure 4E,F shows that MMP7 had higher levels of expression in tumour cells compared to immune cells and normal epithelial cells. In a group of transcriptomics data from TCGA, patients with high levels of MMP7 expression had noticeably lower OS (Figure 4G).

We subsequently examined the spatial expression patterns of MMP7 in samples with two types of immune infiltration (Figure 4H). MMP7 was predominantly expressed in tumour cells rather than normal epithelial cells and immune/stromal cells, consistent with the analysis in single‐cell analysis. In immune exclusion samples, MMP7 showed notable expression within tumour areas, while in samples with immune activation, MMP7 displayed significantly lower expression levels. Furthermore, we conducted IHC on samples from 60 NSCLC patients to delve into the clinical significance of MMP7. Tumour cells exhibited increased expression of MMP7 in comparison to normal epithelial cells, as shown in Figures 4I,J. Furthermore, individuals with advanced NSCLC demonstrated elevated levels of MMP7 expression in comparison to those in the early stages (Figures 4I,J).

### 2.5 The co‐location of MMP7+ tumour cells and CD14+APOE+ cells worsen the clinical outcome of NSCLC patients

**FIGURE 5:**
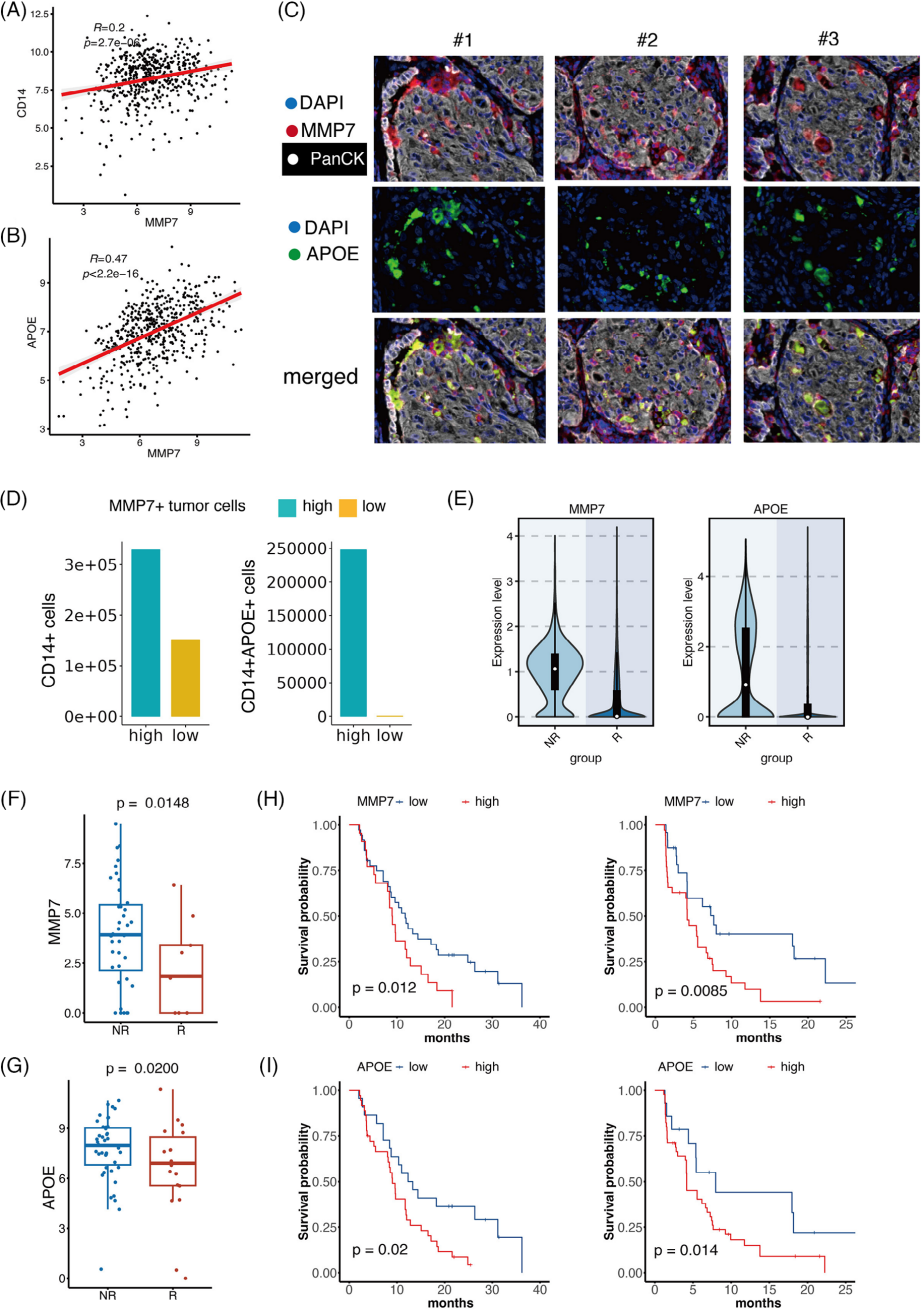
The co‐location of MMP7+ tumour cells and CD14+APOE+ cells worsen the clinical outcome of non‐small cell lung cancer (NSCLC) patients. (A) The correlation of MMP7 and CD14 in the bulk transcriptomics data. (B) The correlation of MMP7 and APOE in the bulk transcriptomics data. (C) The multiplex immunofluorescence performed in 20 NSCLC patients demonstrated the co‐location of MMP7+ tumour cells and CD14+APOE+ cells in NSCLC tumour area. (D) The distribution of CD14+ cells and CD14+APOE+ cells in areas with high/low MMP7+ tumour cells. (E) MMP7 in tumour cells and APOE in myeloid cells had elevated expression in non‐responders (NR) patients than responders (R). (F) The NR exhibited higher MMP7 compared to responders in NSCLC patients treated with immunotherapy. (G) The NR exhibited higher APOE compared to responders in NSCLC patients treated with immunotherapy. (H) Patients with higher MMP7 had significantly worse therapeutic results. (I) Patients with higher APOE had significantly worse therapeutic results.

In order to confirm the presence of CD14+APOE+ cells and MMP7 tumour cells in close proximity, we determined the association between MMP7 and CD14 as well as APOE in a substantial group of NSCLC patients. The connection between MMP7 and CD14 was.2, whereas the correlation between MMP7 and APOE was.47. This suggests that MMP7 is more closely linked to the APOE+ subset of CD14+ cells (Figure 5A,B). In order to confirm this link at the protein level, we performed multiplex immunofluorescence of MMP7 and APOE on 20 samples from patients with NSCLC. Our findings align with the transcriptomics study, as we detected the presence of MMP7+ tumour cells and CD14+APOE+ cells in the NSCLC tumour region (Figure 5C). Moreover, we utilised the software QuPath to perform detailed and quantitative analysis of stained tissue sections. We started by importing the images into QuPath. To determine marker‐positive cells, we set a positive threshold based on the expression level of the marker and the intensity of nuclear expression. A cell is counted as marker‐positive if it meets both of the following criteria: it shows positive expression in the DAPI channel (indicating the presence of a nucleus) and it exceeds the threshold intensity in the marker channel. Next, we manually selected regions of interest (ROIs). Specifically, we selected five ROIs with high MMP7+ tumour cells and five ROIs with low MMP7+ tumour cells in each sample. We then calculated the number of CD14+ cells and CD14+APOE+ cells within these ROIs and compared them between the two types of ROIs. This quantification revealed that areas with high MMP7+ tumour cells had more CD14+ cells (Figure 5D). Notably, CD14+APOE+ cells were predominantly located in areas with high MMP7+ tumour cells, suggesting a strong co‐localisation pattern between MMP7+ tumour cells and CD14+APOE+ cells (Figure 5D).

**FIGURE 6:**
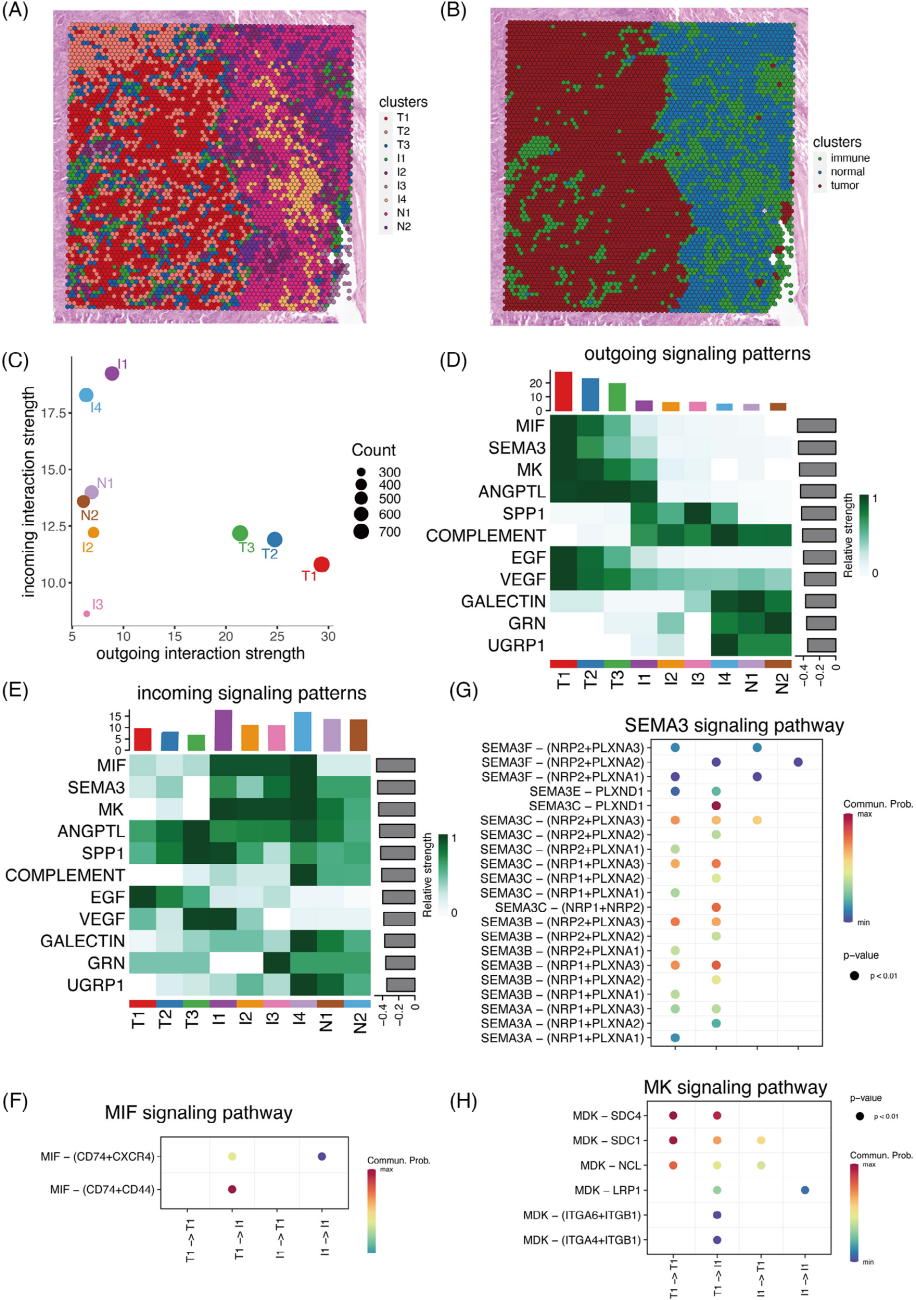
Cell‒cell communication involved in immune exclusion. (A) All spots in p1 were divided into nine clusters, including four immune clusters (I1 to I4), two normal hepatocyte clusters (N1 and N2) and three tumour clusters (T1 to T3). (B) The spatial distribution of immune, tumour and normal clusters. (C) The intensity of cell‒cell communication within all clusters. (D) Overview of the outgoing signalling pathways. (E) Overview of the incoming signalling pathways. (F) The specific cell‒cell interactions in MIF signalling pathway among the tumour and immune clusters. (G) The specific cell‒cell interactions in SEMA3 signalling pathway among the tumour and immune clusters. (H) The specific cell‒cell interactions in MK signalling pathway among the tumour and immune clusters.

Subsequently, we examined the clinical importance of MMP7+ tumour cells and CD14+APOE+ cells. Initially, we examined the clinical implications of these two types of cells in NSCLC patients who underwent EGFR‐TKI therapy. At first, single‐cell data were collected from 49 clinical samples taken from 30 patients with metastatic lung cancer.^16^ These biopsies were obtained before and throughout targeted therapy. The expression of MMP7 in tumour cells and APOE in myeloid cells was higher in non‐responder (NR) patients compared to responders (R) (Figure 5E). Subsequently, we assessed the capacity of tumour cells expressing MMP7 and CD14+ cells expressing APOE to forecast the effectiveness of treatment in patients undergoing immunotherapy. The bulk transcriptomics data from ORIENT‐3 clinical trail (NCT03150875) has been included.^50^ This group comprised 61 patients who had late‐stage NSCLC and experienced treatment failure with first chemotherapy. MMP7 and APOE were both more enriched in NR in patients treated with immunotherapy (Figure 6F,G). We found that patients with higher levels of MMP7 or APOE had significantly worse therapeutic outcomes (Figure 6H,I).

### 2.6 Cell‒cell communication involved in immune exclusion

We then analysed the communication patterns across distinct areas based on ST data. Sample p1, being an immune‐exclusion sample, included diverse areas such as tumour, immune and normal regions. The CellChat analysis on ST data provided a comprehensive landscape of significant cell‒cell communication correlated with immune exclusion. Therefore, we initiated an investigation into the cell‒cell communication between the tumour area and neighbouring immune area in sample p1, aiming to unveil interaction patterns contributing to immune exclusion.

All spots in p1 were divided into nine clusters, including four immune clusters (I1 to I4), two normal epithelial clusters (N1 and N2) and three tumour clusters (T1 to T3) (Figures 6A). The four immune clusters situated near the tumour clusters, while the two normal clusters were positioned slightly further away (Figures 6B). Initially, we examined the intensity of cell‒cell communication within all clusters. The three tumour clusters exhibited the highest level of outgoing interactions, whereas the four immune clusters displayed the highest level of incoming interactions (Figure 6C). These observations indicated that tumour cells play a crucial role in altering the TME to create an immunological exclusion status. Next, we analysed the outgoing and incoming signalling pathways, revealing distinct patterns between the tumour area and normal area. The tumour area activated various proliferation and metastasis‐related signalling pathways (such as MK, SEMA3, MIF, ANGPT, EGF and VEGF) (Figure 6D). On the other hand, the immune clusters were the primary recipients of signalling from the tumour clusters (Figures 6E).

Subsequently, we delved into the specific cell‒cell interactions among the tumour and immune clusters. Our analysis identified the MIF pathway as the primary interaction between tumour and immune clusters. To simplify the presentation of specific signalling pathways, we utilised the T1 in the tumour clusters with the highest outgoing signalling activities and I1 in the immune clusters with the highest incoming signalling activities for further analysis. MIF released from MMP7+ tumour cells activated CD74, CXCR4 and CD44 on immune clusters (Figures 6F). MIF signalling pathway has been identified as a key player in cell‒cell interactions crucial for inflammation and cancer. SEMA3 signalling pathway also showed enhanced activities in the cell‒cell communication (Figures 6G). This signalling pathway consisted of three secreted proteins (SEMA3A, SEMA3B and SEMA3C), which were correlated with tumour metastasis and unfavourable therapy response. Additionally, Midkine (MDK) released from tumour cells interacted with SDC4, SDC1, NCL, ITGA4, ITGA6 and ITGB1 on immune cluster (Figures 6H). MDK are known contributors to critical cancer hallmarks, including cell growth, metastasis and migration.

To ensure that our observations are not limited to a single patient, we have extended our analysis to include a second patient sample (p2). This additional analysis demonstrates similar communication patterns between MMP7+ tumour cells and immune cells, confirming that this phenomenon is not patient specific but rather a broader characteristic of immune exclusion in NSCLC (Figure S2). In addition, focusing on MMP7+ tumour cells could provide a more precise understanding of their specific interactions with immune cells. Therefore, we have reanalysed the single‐cell data to focus specifically on MMP7+ tumour cells. The cell‒cell communication between MMP7+ tumour cells and CD14+APOE+ cells was consistent with the results based on the ST data. This refined analysis confirms that MMP7+ tumour cells exhibit distinct communication patterns with immune cells, particularly through the MIF signalling pathway (Figure S2).

## 3 DISCUSSION

Intra‐tumour immune infiltration plays a pivotal role in influencing anti‐tumour immune responses. This impact on the TME is complex and varies depending on the specific cell types involved. Despite this complexity, investigations into intra‐tumour immune infiltration and its spatial organisation in NSCLC remain limited. This study conducted a thorough analysis of the characteristics and components of immune infiltration in NSCLC. We observed that CD14+APOE+ cells were prominently represented in samples with immune exclusion, exhibiting a strong correlation with MMP7+ tumour cells. Both CD14+APOE+ cells and MMP7+ tumour cells were associated with poorer survival outcomes and unfavourable immunotherapy response, underscoring their potential as biomarkers in NSCLC. Our research provides crucial insights into the intricate patterns of immune cell infiltration within tumours in NSCLC.

Our preliminary studies have revealed clear patterns of immune infiltration in NSCLC, characterised by varying amounts of immune cell presence inside the TME. APOE, an apolipoprotein predominantly recognised for its involvement in cholesterol metabolism, has been linked to several disorders, such as cardiovascular disease and Alzheimer's disease. Functional investigations have demonstrated that exosomes generated from M2 macrophages transfer APOE to cancer cells, resulting in the reduction of MHC‐I expression and inhibition of the tumour's inherent ability to provoke an immune response.^51^ Administering APOE ligand can improve the effectiveness of immunotherapy in cancers that are abundant with M2‐CD14+ cells. Therefore, APOE appears to be a promising indicator and treatment target for immunotherapy resistance in malignancies that are enriched with M2 cells. Furthermore, a significant abundance of APOE+CTSZ+CD14+ cells, which exhibit immunosuppressive properties by increasing the expression of anti‐inflammatory genes, has been detected in a substantial number of tumour specimens, specifically in cases of colorectal cancer.^52^ There is a favourable correlation between the proportion of APOE+CTSZ+CD14+ cells and regulatory T cells in colorectal cancer samples. In clear cell renal cell carcinoma, the existence of C1Q+APOE+ macrophages is associated with tired T cells, creating an impaired immune system in the form of a malfunctioning circuit.^53^ These findings emphasise the importance of APOE in regulating immune responses in the TME and highlight its potential as a target for therapy to overcome resistance to immunotherapy and restore sensitivity to immunotherapy in cancer patients.

In addition, our study examined the possible characteristics of tumours that cause the invasion of CD14+APOE+ cells. MMP7 showed elevated expression levels correlated with the invasion of CD14+APOE+ cells. Evaluating the level of MMP7 expression in fine‐needle aspiration biopsy samples after diagnosis can help identify individuals with surgically removed pancreatic ductal adenocarcinoma who would benefit the most from neoadjuvant therapy.^54^ IHC staining demonstrated a substantial increase in MMP7 expression in 90 NSCLC tissues and cells compared to 50 normal lung tissues.^55^ This higher MMP7 expression was found to enhance the proliferation and invasion of cancer cells.^55^ Furthermore, there was a notable rise in the expression of MMP7 in NSCLC patients who had lymph node metastases, in comparison to healthy tissues.^56^ The levels of MMP7 expression were also found to be linked to the survival of the patients. The results emphasise the significant regulatory functions of MMP7 in NSCLC and indicate its potential as a target for clinical diagnostics and treatment in the future. Moreover, the increased expression of MMP7 can enhance the metastatic properties of colorectal cancer cells via activating the MAPK pathway.^57^ Members of the MMP family have been recognised as therapeutic targets and prognostic biomarkers in the TME of head and neck squamous cell carcinoma. This underscores their potential importance in the treatment of this form of cancer.^58^

In addition, our investigation revealed the geographical co‐localisation of CD14+APOE+ cells and MMP7+ tumour cells, and examined the intercellular communication between these two cell types. The MIF signalling pathway has been recognised as a pivotal factor in cell‒cell interactions that are essential for both inflammation and malignancy. Researchers have discovered that breast cancer stem cells release MIF, which plays a role in tumour metabolic reprogramming that leads to immune evasion.^59^ Reducing MIF levels in breast cancer cells increased the presence of cytolytic CD8+ T cells and pro‐inflammatory macrophages inside the TME, while lowering the number of regulatory T cells and tumour‐associated neutrophils. MIF functions as a 3′ flap nuclease, aiding in DNA replication and stimulating tumour growth.^60^ Furthermore, there is a negative correlation between the amount of MIF and the effectiveness of PD‐1 blockade immunotherapy when used in conjunction with chemotherapy as a neoadjuvant treatment for esophageal squamous cell carcinoma.^61^ In addition, MIF has been recognised as a crucial controller of the infiltration of mononuclear phagocytes in hepatocellular carcinoma. Furthermore, it has been discovered that CD36+ cancer‐associated fibroblasts contribute to the creation of an immunosuppressive milieu for hepatocellular carcinoma by releasing MIF.^62^, ^63^

MMP7, also known as matrilysin, is a matrix metalloproteinase that plays a crucial role in the degradation of the ECM. It is involved in various aspects of cancer progression, including tumour growth, invasion, metastasis and angiogenesis. MMP7 is known to be overexpressed in many types of cancer and is associated with poor prognosis. APOE+ Tumor‐associated macrophages (TAMs) are a subset of macrophages that exhibit immunosuppressive properties and are known to promote tumour progression. These TAMs can enhance the invasive potential of cancer cells by secreting various cytokines and proteases, including MMPs. In our study, we observed co‐localisation of APOE+ TAMs and MMP7+ cancer cells in the TME. This spatial proximity suggests a potential interaction where APOE+ TAMs may contribute to the upregulation of MMP7 in cancer cells, thereby facilitating tumour invasion and metastasis. APOE+ TAMs may secrete factors that induce MMP7 expression in nearby cancer cells, enhancing their invasive capabilities.

Our study primarily focuses on analysing human NSCLC samples to provide direct relevance to clinical outcomes. The use of human samples allows us to investigate the TME and immune infiltration patterns within the context of actual patient biology and treatment responses. This approach ensures that our findings are immediately applicable to clinical settings. The innovative use of ST provides high‐resolution spatial and molecular data that is challenging to replicate in animal models. This technology enables us to capture the complex spatial interactions and cellular heterogeneity within human tumours, offering insights that are directly translatable to patient care. The inclusion of multiplex immunofluorescence further validates our findings within human tissues.

We recognise the value of animal experiments for mechanistic validation and functional studies. In future work, we plan to extend our research by incorporating animal models to experimentally validate the interactions between CD14+APOE+ cells and MMP7+ tumour cells. These experiments will aim to confirm the causal relationship between the co‐location of CD14+APOE+ cells and MMP7+ tumour cells and immune resistance mechanisms, investigate the specific signalling pathways (MK, SEMA3 and MIF) identified in our study within a controlled animal model, and test potential therapeutic interventions targeting these interactions to assess their efficacy in overcoming immune resistance. We are currently in the process of designing animal studies to further explore the functional implications of our findings. These studies will include creating genetically engineered mouse models to express APOE and MMP7 in a spatially controlled manner, conducting in vivo imaging and single‐cell RNA sequencing to observe the dynamics of immune cell interactions within the TME, and evaluating the impact of disrupting the CD14+APOE+ cells and MMP7+ tumour cell interaction on tumour growth and response to immunotherapy.

Despite the comprehensive nature of this study, several limitations should be acknowledged. First, further validation in independent cohorts and prospective studies is necessary to confirm clinical utility of CD14+APOE+ cells and MMP7+ tumour cells. Second, while our spatial analysis revealed the co‐location of CD14+APOE+ cells with MMP7+ tumour cells, the functional implications of this co‐localisation were inferred based on known roles of these cell types and their expression profiles. Experimental validation, such as functional assays or in vivo models, is required to definitively establish the mechanistic interactions between CD14+APOE+ cells and MMP7+ tumour cells in promoting immunotherapy resistance. Lastly, while we used multiplex immunofluorescence to validate the presence of CD14+APOE+ cells and MMP7+ tumour cells, additional validation using other complementary techniques such as flow cytometry or single‐cell RNA sequencing could provide a more robust confirmation of our findings. In summary, while our study offers significant insights into the spatial structure and functional characteristics of CD14+APOE+ cells and MMP7+ tumour cells in NSCLC, additional research is needed to address these limitations and to further elucidate the mechanisms underlying therapy resistance.

## 4 MATERIALS AND METHODS

### 4.1 Patient samples

The ethics committee of Cancer Hospital, Chinese Academy of Medical Sciences (No. 23/262‐4004) approved this study, which adhered to the principles of the Declaration of Helsinki. Written informed consent was obtained from all participants in our hospital who donated surgical tissues. Qualified pathologists confirmed all diagnoses through histological reviews.

### 4.2 Data and materials

The single‐cell data from GSE148071, GSE127465 and GSE131907 were acquired from the Gene Expression Omnibus database. The relevant clinical data were obtained from the original research. Furthermore, we collected single‐cell data from 49 clinical samples taken from 30 patients with metastatic lung cancer before and during targeted therapy with EGFR‐TKI. The original study included information on the response status of these patients.

### 4.3 Spatial transcriptomics sequencing

FFPE human cancer specimens were acquired from patients. The sequencing procedure was conducted following the Visium Spatial Gene Expression for FFPE reagent kit technique provided by 10× Genomics firm (User Guide CG000407 Rev C, human transcriptome product number 1000338). This platform supplies the reagents and consumables required for the experiment. The specific product numbers may be obtained at www.10xgenomics.com/products/spatialgene‐expression.

### 4.4 Pathological annotations for HE images

Within the scope of this investigation, pathologists were responsible for annotating the macroscopic tissue appearance, as well as the histological and clinicopathological findings of the tumour tissues. All of the spots were separated into different histological categories, which included normal epithelial cells, tumour cells, fibroblasts, endothelial cells and immune cells.

### 4.5 Clustering analysis of spatial transcriptomics

Space Ranger was used to generate gene‐spot matrices, which then underwent initial routine statistical analysis. This included estimating the number of identified Unique molecular identifiers (UMIs) (nUMI) and genes (nGene) for each spot. We eliminated spots with exceptionally low nUMI or nGene values, as well as patches located away from the main tissue sections. Additionally, genes expressed in fewer than three locations, along with mitochondrial and ribosomal genes, were excluded. The ST data were then subjected to clustering analysis using Seurat, with parameters carefully adjusted to ensure optimal cell type classification. We then employed the SCTransform function to normalise the count data. We subsequently identified 3000 genes with significant variability based on their average expression levels and variances. Using these genes, we conducted principal components analysis (PCA) to map the spots onto a low‐dimensional space defined by the first 30 principal components (PCs). This rectified PC matrix was then used for unsupervised clustering and uniform manifold approximation and projection (UMAP) visualisation analysis based on the shared‐nearest neighbour algorithm.

### 4.6 Identification of malignant cells in spatial analysis

For distinguishing malignant from non‐malignant spots, hierarchical clustering using the inferCNV package with the random trees method was utilised. The tumour cluster was determined based on CNV scores and pathological annotations. The detailed method could be found in our previous study.

### 4.7 Differential expression analysis and gene set enrichment analysis

We utilised the FindAllMarker function to detect genes that exhibit differential expression between clusters. The parameters employed were min.pct = .1 and logfc.threshold = .25. The non‐parametric Wilcoxon rank‐sum test was utilised to calculate *p*‐values for comparisons, and the corrected *p*‐values, employing Bonferroni correction, were obtained for all genes in the dataset. To elucidate the biological functions of the identified DEGs in each cluster, we performed gene set enrichment analysis. The Kyoto Encyclopedia of Genes and Genomes, cancer hallmark signatures and Biological Process Gene Ontology were analysed.

### 4.8 Dimension reduction and clustering analysis for single‐cell data

We utilised the FindVariableFeatures function in the R package Seurat to scale the data using the top 2000 genes with the most variability. We employed variable genes for PCA, utilised the FindNeighbors function in Seurat to determine the nearest neighbours for graph clustering based on PCs, employed the FindCluster function in Seurat to identify cell subtypes, and visualised the cells using the UMAP algorithm. In order to mitigate the batch impact, we utilised the Harmony algorithm from the Harmony R package to do batch correction. Additionally, we employed the FindNeighbors and FindCluster functions in Seurat to identify cell subtypes. The cells were labelled using carefully selected markers, such as EPCAM, KRT8 and KRT18 for epithelial cells, COL1A1, COL6A1 and FAP for fibroblasts, PLVAP, VWF and PECAM1 for endothelial cells, CD68, CD14, XCR1, CLEC9A and CLEC10A for myeloid cells, NKG7, KLRC1, CCR7, CD8B and CD3D for T cells, and MZB1, CD19 and AICDA for B cells.

### 4.9 Transcription factor analysis

The DoRothEA database includes the information of TFs and their interactions with target genes. Our analysis includes interactions with confidence levels A, B and C inside the database. The run_viper function was used to compute the activities of the regulons. We integrated the VIPER algorithm with TFs data to accurately assess TF activity. Moreover, the ‘FindAllMarkers’ function was employed to detect the leading TFs for each cluster, arranged in order of log2 fold change.

### 4.10 Multiplex immunofluorescence

The multiplex immunofluorescence panel, comprised of panCK (abcam, ab234297), MMP7 (abcam, ab207299), APOE (abcam ab183597) and CD14 (CST D7A2T), was conducted following the manufacturer's instructions provided by Akoya's 4‐Colour Multiple IHC Kit.

### 4.11 IHC analysis

A total of 60 NSCLC samples underwent IHC analysis. The tissue slices were initially dewaxed and then subjected to overnight incubation at 4°C with specific primary antibodies (MMP7 abcam, ab207299). Each sample was assigned a score based on the intensity of staining (0 = no staining; 1 = weak staining; 2 = moderate staining; and 3 = strong staining) and the proportion of stained cells (0 = 0%; 1 = 1%−25%; 2 = 25%−50%; 3 = 50%−75%; and 4 = 75%−100%). The final score was calculated as the product of the staining intensity and positive area score, resulting in a range from 0 to 12. The IHC results of the tissues were independently reviewed by two experienced pathologists who were blinded to the clinical parameters, ensuring the accuracy and reliability of the analyses.

### 4.12 Survival analysis

For the survival studies, patient samples were divided into two groups, high and low, based on gene expression levels. The log‐rank test was utilised to determine the disparity between the survival curves of the two groups. Afterwards, the ggsurvplot tool was used to create Kaplan‒Meier survival curves in order to display the survival results of the two expression groups.

### 4.13 Data of patients treated with immunotherapy from ORIENT‐3 study

The ORIENT‐3 trial was a phase 3 clinical study (NCT03150875). Approval from the Ethics Committee was received from all participating centres, and written informed permission was obtained from all patients. The main outcome measure was OS, which was defined as the time from randomisation to death from any cause within the whole study set. Patients who received anti‐PD‐1/PD‐L1 medication before their disease progressed after being randomly assigned to the docetaxel treatment group were not included in the whole analysis set. Sixty‐one samples from the sintilimab group were included in the downstream analysis. RNA was isolated from FFPE baseline tumour tissues using the RNeasy FFPE Kit (Qiagen).

### 4.14 Cell‒cell communication analysis

The CellChat package was employed to deduce, examine and depict intercellular communication relationships among cell subsets at both the single‐cell and ST levels. At first, we determined the ligands or receptors that were expressed at higher levels in each cell type. Then, we estimated the likelihood of communication by analysing all the interactions between ligands and receptors that are linked to each signalling pathway.

### 4.15 Statistical analysis

The Mann‒Whitney *U*‐test was employed to analyse the differences between the two groups and the Kruskal‒Wallis rank sum test was applied to calculate statistical differences from multiple groups using the R package ‘stats’. The Spearman's correlation test was utilised to assess the correlations between two variables. A significance threshold of.05 (two‐tailed) was applied for determining statistical significance.

## AUTHOR CONTRIBUTIONS

*Conception/design*: Yuankai Shi and Xiaohong Han. *Methodology*: Guangyu Fan, Le Tang and Tongji Xie. *Formal analysis*: Guangyu Fan, Lin Li and Tongji Xie. *Writing—manuscript writing*: Guangyu Fan, Tongji Xie and Le Tang. *Writing—revising and editing*: Yuankai Shi and Xiaohong Han.

## CONFLICT OF INTEREST STATEMENT

The authors declare they have no conflicts of interest.

## ETHICS STATEMENT

All samples were collected with the approval of the ethics committee of the Cancer Hospital of the Chinese Academy of Medical Sciences (No. 23/262‐4004) and following the principles outlined in the Declaration of Helsinki.

## Supporting information

Supporting information

## Data Availability

The raw sequence data reported in this paper have been deposited in the Genome Sequence Archive in National Genomics Data Center, China National Center for Bioinformation/Beijing Institute of Genomics and Chinese Academy of Sciences (GSA:HRA006757).
