# Supplementary material for: The co‐location of CD14+APOE+ cells and MMP7+ tumour cells contributed to worse immunotherapy response in non‐small cell lung cancer
Source: Clin Transl Med. 2024 Aug 26;14(9):e70009. doi: 10.1002/ctm2.70009 (PMC11347392; doi:10.1002/ctm2.70009)
Supplement: Supplementary file 1 — Supporting information [file CTM2-14-e70009-s001.docx]

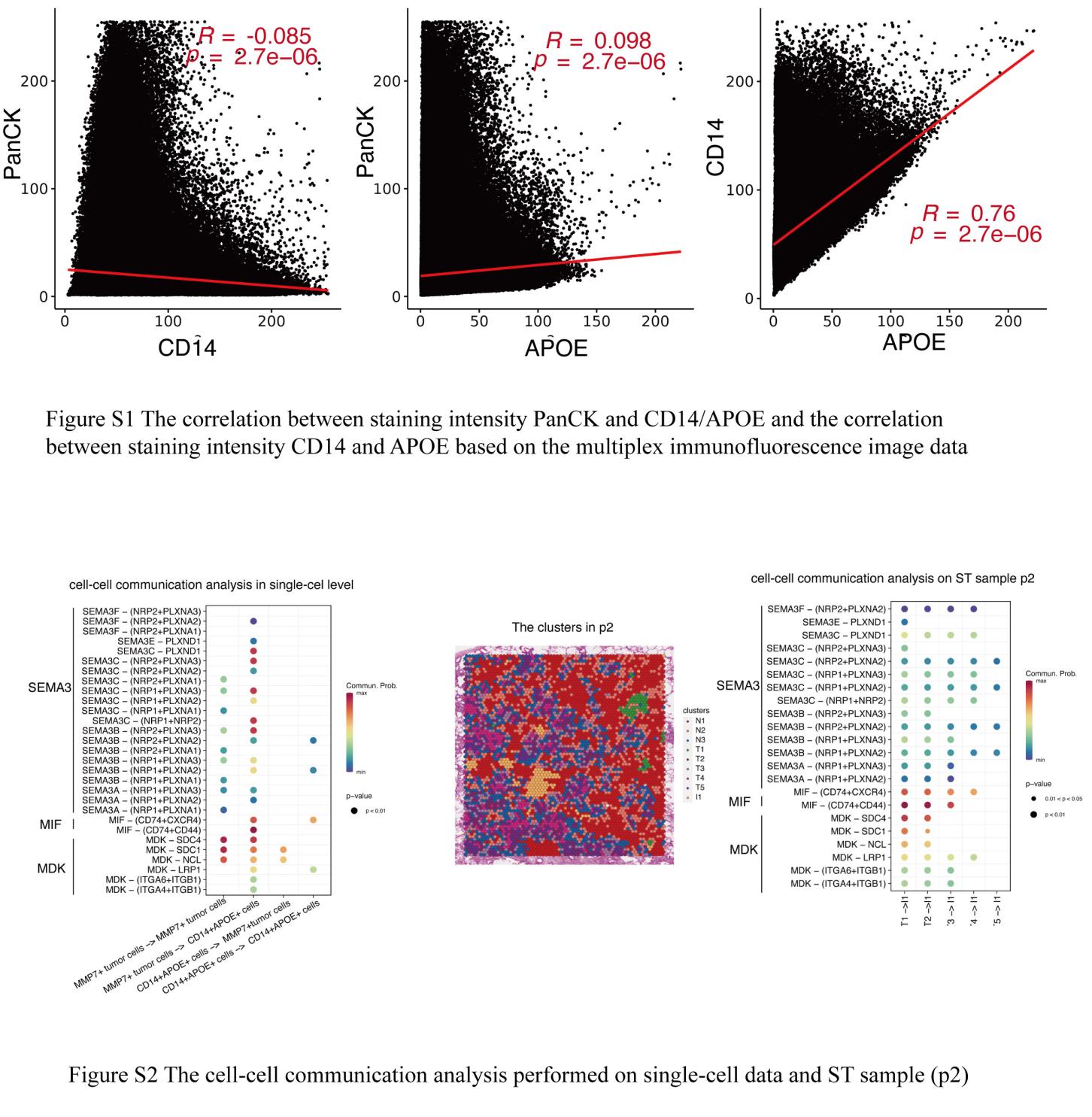


Table S1: The The detailed quality information of 6 ST samples.

| sample | Mean Reads per Spot | Mean Reads Under Tissue per Spot | Fraction of Spots Under Tissue | Reads Mapped to Probe Set | Reads Mapped Confidently to Probe Set | Fraction Reads in Spots Under Tissue | Median Genes per Spot | Median UMI Counts per Spot | Genes Detected |
| --- | --- | --- | --- | --- | --- | --- | --- | --- | --- |
| p1 | 71070.03 | 70253.86 | 1 | 0.98 | 0.98 | 1 | 4566.5 | 10002 | 18040 |
| p2 | 74092.74 | 72944.5 | 0.94 | 0.99 | 0.98 | 0.99 | 7138.5 | 30512.5 | 18059 |
| p3 | 64385.89 | 63747 | 0.99 | 0.99 | 0.99 | 1 | 7729.5 | 38078.5 | 18047 |
| p4 | 69052.37 | 68482.48 | 1 | 0.99 | 0.98 | 1 | 5697.5 | 14317 | 18062 |
| p5 | 70420.55 | 69833.25 | 1 | 0.99 | 0.85 | 1 | 3544.5 | 8679.5 | 18072 |
| p6 | 72496.57 | 71287.92 | 0.9 | 0.99 | 0.98 | 0.99 | 5199.5 | 11014 | 18050 |

Table S2: The top 50 differentially expressed genes of intra-tumor immune clusters in all samples.

| P1 | P2 | P3 | P4 | P5 | P6 |
| --- | --- | --- | --- | --- | --- |
| CD14 | APOE | IGHA1 | IGHG1 | IGKC | IGHG1 |
| APOE | CD14 | IGKC | IGHG3 | IGHG3 | IGHG3 |
| IFI30 | APOC1 | IGHG1 | JCHAIN | JCHAIN | SCGB3A2 |
| APOC1 | CTSB | SCGB3A2 | IGHA1 | IGHG1 | JCHAIN |
| CHIT1 | IFI30 | JCHAIN | TIMP1 | IGLC1 | IGLC1 |
| DCN | SFTPA1 | IL7R | CD4 | IGHA1 | IGHM |
| CTSE | VIM | CD3 | MS4A6A | TRAC | CCL22 |
| LYZ | HBA2 | SRGN | TRBC2 | C1QC | CD74 |
| CCND2 | CXCL5 | CD2 | IL7R | CXCR4 | GPNMB |
| LAPTM5 | COL3A1 | MS4A6A | C1QC | LAPTM5 | IGHD |
| C1QB | COL1A1 | LYZ | C1QA | LCP1 | C1QC |
| CD74 | JCHAIN | CTSE | TRAC | MS4A6A | CTSB |
| CLDN1 | LYZ | MS4A7 | LCP1 | COL1A1 | SFRP4 |
| C1QC | DCN | SPINK1 | CCL20 | COL6A3 | RGS1 |
| SMC4 | A2M | CTSZ | CTSB | CTHRC1 | C1QA |
| GPX2 | HMOX1 | CD4 | LSP1 | FCGBP | COL3A1 |
| MMP1 | COL6A3 | IFI30 | SPP1 | IL7R | C1QB |
| NRARP | RGS1 | LCP1 | JCHAIN | CD2 | COL1A2 |
| PSME1 | LUM | CD53 | SRGN | COL1A2 | POSTN |
| PSAP | IGHM | IKZF1 | CD2 | THBS2 | SRGN |
| NME2 | IGHG1 | CCL22 | FCGBP | CCL19 | CD68 |
| MMP13 | COL1A2 | HCLS1 | S100A6 | CD3 | FCGBP |
| CTSC | C1R | CXCR4 | C1QB | POSTN | MS4A6A |
| LMNB2 | C1S | IL32 | MS4A6A | CCL20 | LYZ |
| UBE2C | SERPINE1 | B2M | CCL22 | CD4 | CTSE |
| CCND1 | CXCR4 | TMEM176B | SPI1 | SFRP2 | CTHRC1 |
| TXNDC17 | SRGN | TYROBP | CXCR4 | LYZ | CTSZ |
| C7orf50 | IL7R | SAMHD1 | FTL | F13A1 | F13A1 |
| EFHD2 | SFTPC | AIF1 | GPNMB | SFRP4 | FCER1G |
| CTSZ | MT2A | GRN | CYBB | CCL22 | LAPTM5 |
| TRUB1 | EGR1 | S100A6 | CD53 | CCL19 | COL1A1 |
| TIMM10 | COL6A2 | GPX2 | CCL19 | AIF1 | CXCR4 |
| PHLDA2 | LGALS1 | IFI6 | MS4A7 | SYNGR2 | FTL |
| FAM49B | DMBT1 | GCNT3 | AIF1 | ARHGDIB | COL6A3 |
| DAB2IP | ISG15 | TSPAN8 | HCLS1 | STAT1 | MMP9 |
| SLC6A15 | SPARC | TM4SF4 | IGHA1 | S100A6 | CCL20 |
| SMARCA4 | IGFBP7 | CTSB | PSAP | GRN | LSP1 |
| COX8A | B2M | GRN | SAMHD1 | CLDN4 | CSF1R |
| PSMB9 | CTSL | FCER1G | CORO1A | B2M | TMEM176B |
| MYC | PTGDS | TESC | IGLC1 | CTSZ | IFI30 |
| PPA1 | RGCC | TOB1 | B2M | IL32 | MAFB |
| CDK6 | TGM2 | ANXA4 | LTB | CTSS | CXCL9 |
| CIB1 | SFTPD | CYP27A1 | CTSZ | IGHM | B2M |
| CCT6A | FN1 | CTSS | CAPG | IGHD | IL4I1 |
| GPNMB | GPNMB | ISG15 | IL7R | CTSL | CCL19 |
| ALG3 | GPX3 | IER3 | TYROBP | LITAF | CYBB |
| FTL | IKZF1 | SOX9 | TMEM176B | CTSL | AIF1 |
| SAT1 | MMP2 | IL4I1 | IKZF1 | COL1A2 | THBS2 |
| NPW | CTSC | THBS2 | IL32 | LGMN | SFRP2 |
| MT2A | CTSZ | CTSL | SFTPD | ACTB | LCP1 |

Table S3: The top 50 differentially expressed genes of tumor area in samples with distinct immune infiltration patterns.

| cluster | gene | cluster | gene |
| --- | --- | --- | --- |
| Immune activation | SFN | Immune exclusion | MMP7 |
| Immune activation | SCGB3A1 | Immune exclusion | COL17A1 |
| Immune activation | SCGB3A2 | Immune exclusion | VEGFA |
| Immune activation | IGHG3 | Immune exclusion | IGFL2 |
| Immune activation | PIGR | Immune exclusion | SLC6A14 |
| Immune activation | MT-ND3 | Immune exclusion | GJA1 |
| Immune activation | MSLN | Immune exclusion | C15orf48 |
| Immune activation | SLPI | Immune exclusion | FHL2 |
| Immune activation | SPINK1 | Immune exclusion | CYP24A1 |
| Immune activation | WFDC2 | Immune exclusion | S100A2 |
| Immune activation | SFTPC | Immune exclusion | MT2A |
| Immune activation | GLB1L3 | Immune exclusion | SPRR1B |
| Immune activation | HHIP | Immune exclusion | FAM83A |
| Immune activation | CTSH | Immune exclusion | PMAIP1 |
| Immune activation | CYP4B1 | Immune exclusion | TMPRSS11E |
| Immune activation | LRRK2 | Immune exclusion | GLS |
| Immune activation | MGP | Immune exclusion | SLC2A1 |
| Immune activation | TPPP3 | Immune exclusion | PLAU |
| Immune activation | PTPN13 | Immune exclusion | IL37 |
| Immune activation | C16orf89 | Immune exclusion | SEMA3B |
| Immune activation | ABCA3 | Immune exclusion | TNNT1 |
| Immune activation | PGC | Immune exclusion | TXNRD1 |
| Immune activation | C3 | Immune exclusion | VSIG1 |
| Immune activation | EPHX1 | Immune exclusion | UBE2C |
| Immune activation | C1orf116 | Immune exclusion | TMSB10 |
| Immune activation | TXNIP | Immune exclusion | SOX4 |
| Immune activation | CYBRD1 | Immune exclusion | TNFRSF21 |
| Immune activation | NAPSA | Immune exclusion | ANXA1 |
| Immune activation | MUC1 | Immune exclusion | KRT7 |
| Immune activation | TFPI2 | Immune exclusion | ISG15 |
| Immune activation | SFTPB | Immune exclusion | PYGL |
| Immune activation | SPP1 | Immune exclusion | SEMA4B |
| Immune activation | STEAP4 | Immune exclusion | KRT19 |
| Immune activation | SFTPA1 | Immune exclusion | WSB1 |
| Immune activation | SELENBP1 | Immune exclusion | RNF19A |
| Immune activation | IGKC | Immune exclusion | MYEOV |
| Immune activation | HABP2 | Immune exclusion | OAS3 |
| Immune activation | PARM1 | Immune exclusion | EREG |
| Immune activation | FOLR1 | Immune exclusion | AZGP1 |
| Immune activation | C4BPA | Immune exclusion | ITPKA |
| Immune activation | IGHM | Immune exclusion | CCL20 |
| Immune activation | PPP1R1B | Immune exclusion | NAMPT |
| Immune activation | SCGB1A1 | Immune exclusion | FAM3C |
| Immune activation | CD74 | Immune exclusion | AREG |
| Immune activation | TMPRSS2 | Immune exclusion | AHNAK2 |
| Immune activation | FAM3B | Immune exclusion | ERO1A |
| Immune activation | KIAA1324L | Immune exclusion | OAS1 |
| Immune activation | CLU | Immune exclusion | CCND1 |
| Immune activation | VWA2 | Immune exclusion | IGFBP2 |
| Immune activation | SERPIND1 | Immune exclusion | GJA1 |
